# Supplementary material for: Time in Range in Children with Type 1 Diabetes before and during a Diabetes Camp—A Ceiling Effect?
Source: Children (Basel). 2022 Dec 12;9(12):1951. doi: 10.3390/children9121951 (PMC9777517; doi:10.3390/children9121951)
Supplement: Supplementary file 1 [file children-09-01951-s001.zip › children-1999701-supplementary.pdf]

SUPPLEMENT

Suppl. Table S1. Selection of participants

Total number of camp participants	38
Number of camp participants without CGM pre-camp and camp	-6
Number of camp participants with short pre-camp record time	-4
Number of camp participants with short camp record time	-2
Total number of camp participants included	26

Suppl. Table S2. Glycemic Control before and during camp

		Difference between insulin pen and pump		
	N	Insulin pen<br>10	Insulin pump<br>16	p-value<br>.
Time on record (days)	pre-camp<br>camp	.<br>.	.<br>.	.<br>.
Time on record (%)	pre-camp<br>camp	96 [84;100]<br>97 [96;98]	94 [80;96]<br>95 [89;97]	0.37<br>**0.045**
Time in range (TIR) (%)	pre-camp<br>camp	57.7 ± 21.4<br>67.6 ± 14.2	58.5 ± 15.2<br>61.4 ± 10.8	0.91<br>0.82
Time below range (TBR) (%)	pre-camp<br>camp	4.0 ± 3.1<br>5.4 ± 3.1	2.7 ± 1.6<br>5.5 ± 3.1	0.16<br>0.92
Time above range (TAR) (%)	pre-camp<br>camp	38.4 ± 19.6<br>27.0 ± 12.7	38.9 ± 15.6<br>27.9 ± 8.9	0.94<br>0.83
Daytime TIR (%)	pre-camp<br>camp	55.7 ± 21.8<br>64.3 ± 15.9	55.4 ± 16.8<br>61.4 ± 10.8	0.96<br>0.58
Nighttime TIR (%)	pre-camp<br>camp	60.9 ± 22.0<br>73.0 ± 14.7	63.6 ± 14<br>74.8 ± 6.7	0.70<br>0.68
Daytime TBR (%)	pre-camp<br>camp	2.8 [1.1; 6.3]<br>2.4 [2.1; 5.7]	2.1 [0.8; 2.6]<br>4.1 [2.7; 6.1]	0.34<br>0.17
Nighttime TBR (%)	pre-camp<br>camp	4.7 ± 4.1<br>8.7 ± 5.5	3.4 ± 2.5<br>6.9 ± 4.7	0.33<br>0.40
Daytime TAR (%)	pre-camp<br>camp	40.7 ± 20.2<br>32.4 ± 15.6	42.4 ± 18.0<br>34.0 ± 11.7	0.83<br>0.76
Nighttime TAR (%)	pre-camp<br>camp	34.4 ± 20.0<br>18.3 ± 10.9	33.1 ± 13.4<br>18.3 ± 7.9	0.84<br>0.99
Scans per day with isCGM	pre-camp<br>camp	13.1 ± 5.7<br>16.7 ± 5.3	13.1 ± 3.7<br>19.7 ± 4.0	0.98<br>0.26
Difference TIR pre-camp to camp	(%)	10.0 ± 9.3	8.2 ± 16.7	0.75
Difference TBR pre-camp to camp	(%)	1.4 ± 1.4	2.8 ± 2.8	0.14
Difference TAR pre-camp to camp	(%)	-11.4 ± 9.3	-11.0 ± 17.8	0.95
Difference TDD pre-camp to camp	(%)	-17.3 ± 13.5	-19.0 ± 9.6	0.71
In case of normal distribution, data is given as mean ± standard deviation and t-test was used for comparison between groups and paired t-test for comparison pre-camp and camp. For quantitative data without normal distribution Mann-Whitney-U-Test and Wilcoxon Ranks Test were used for comparison and data are given as median [25th;75th percentile].				

Suppl. Table S3: Technology Use

			Insulin application		Pump models		
			Pen	Pump	AccuChek Insight	Omnipod	Medtronic 640G
**all CGM Systems**		26	10 (38.5%)	16 (61.5%)	1 (3.8%)	3 (11.5%)	12 (46.2%)
CGM System	rtCGM	13 (50%)	3 (11.5%)	10 (38.4%)	.	1 (3.8%)	9 (34.6%)
	isCGM	13 (50%)	7 (26.9%)	6 (23.1%)	1 (3.8%)	2 (7.7%)	3 (11.5%)
CGM Devices	Medtronic Enlite	9 (35%)	.	9 (34.6%)	.	.	9 (34.6%)
	Dexcom G6	4 (15%)	3 (11.5%)	1 (3.8%)	.	1 (3.8%)	.
	Abbott Freestyle Libre	13 (50%)	7 (26.9%)	6 (23.1%)	1 (3.8%)	2 (7.7%)	3 (11.5%)
Data is n (%).							
